# Health management strategies of resilient honey bee stock throughout Southern California

**DOI:** 10.1093/jee/toag099

**Published:** 2026-04-25

**Authors:** Genesis Chong-Echavez, Jessica Webb, Boris Maciejovsky, Boris Baer

**Affiliations:** Department of Entomology, Center for Integrative Bee Research, University of California, Riverside, Riverside, CA, USA; Department of Entomology, Center for Integrative Bee Research, University of California, Riverside, Riverside, CA, USA; Department of Entomology, Center for Integrative Bee Research, University of California, Riverside, Riverside, CA, USA; School of Business, University of California, Riverside, Riverside, CA, USA; Department of Entomology, Center for Integrative Bee Research, University of California, Riverside, Riverside, CA, USA

**Keywords:** apiculture, beekeeping, management practice, integrated pest management, economics

## Abstract

Significant losses of managed honey bees (*Apis mellifera*) have been documented in recent years, driven by multiple stressors including parasites, pesticide exposure, and environmental change. To mitigate escalating hive losses, some beekeepers in Southern California collect and propagate locally occurring, free-living colonies, often referred to as “survivor” or “Californian” honey bees, that persist with limited human intervention. We conducted a cross-sectional survey of beekeepers managing colonies in Southern California to compare management practices and reported outcomes across stock types. Beekeepers clustered into 3 groups: those managing only commercial colonies (35.3%), only Californian colonies (29.4%), or a mixture of both (35.3%). Respondents indicated that management practices had been adapted when keeping Californian honey bees and reported reduced expenditures associated with queen replacement and disease management. These bees were widely perceived to possess beneficial traits, including greater colony persistence and potential climate resilience. However, quantitative analyses did not detect significant differences in reported parasite prevalence among stock types, highlighting the importance of distinguishing perception from measured outcomes. Additional studies are needed to evaluate characteristics such as defensiveness, productivity, and seasonal survival using standardized approaches. Overall, our findings suggest that Californian stock may represents a viable and potentially cost-effective component of sustainable apiculture. Further research is required to clarify the ecological, genetic, and physiological mechanisms underlying beneficial key life-history traits, which may involve tolerance, resistance, or their interaction. Such knowledge would provide a foundation for future breeding efforts aimed at developing honey bee populations that are robust to multiple ecological stressors.

## Introduction

The availability of honey bees [*Apis mellifera* Linnaeus {Hymenoptera: Apidae}] for recreational and pollination purposes is critical, yet beekeepers in the United States face increasing challenges in maintaining healthy colonies. Since 2011, annual colony losses in the United States have averaged 30% to 40% (Spleen et al. 2013, Kulhanek et al. 2017). Recent estimates reported losses exceeded 60%, the highest level recorded to date for commercial honey bees (Lamas et al. 2024). The decline in honey bee health has been extensively studied. Multiple drivers of colony losses have been identified (vanEngelsdorp et al. 2009), including climatic and environmental change (Le Conte and Navajas 2008, Vasiliev and Greenwood 2021), the spread of diseases and parasites (Goulson et al. 2015), nutritional deficiencies (Naug 2009), and pesticide exposure (Al Naggar and Baer 2019, Tosi et al. 2021).

Beekeepers have domesticated honey bees for at least 7,000 years (Bloch et al. 2010) to produce honey and other bee products (Roffet-Salque et al. 2015). The industrialization of agriculture now requires large numbers of colonies to pollinate more than 80 crops of agricultural importance (Tubene et al. 2023). This demand has driven large-scale queen rearing and colony management (Cobey et al. 2011), with breeding programs selecting for traits of commercial relevance, such as pollination efficiency and reduced defensiveness (Schneider et al. 2004). However, these activities have also led to a documented reduction in the genetic diversity of commercial bee stocks in the United States (Cobey et al. 2011). Genetic diversity at both the individual and colony levels influence performance and disease tolerance, and its loss has been linked to documented declines in colony health (Tarpy and Seeley 2006, Mattila and Seeley 2007, Tarpy et al. 2013, Delaplane et al. 2021).

In response to ongoing declines, some beekeepers have begun sourcing colonies from locally adapted populations rather than relying solely on commercial US stocks (Zárate et al. 2023a, 2020b). These locally occurring honey bees persist without intensive human intervention and suggest they possess life-history traits shaped by natural selection that support persistence under parasite pressure and climatic stress. Southern California harbors such a population of “survivor” honey bees, which we refer to here as “Californian honey bees” to distinguish this stock from commercial populations and other bees in the region (Chong-Echavez and Baer 2026). Genetic analyses confirm that this population is admixed, with ancestry from Western European, Eastern European, Middle Eastern, and African lineages (Branchiccela et al. 2014, Zárate et al. 2022, Allen, personal observation). Hybridization appears to be ongoing, as commercial colonies transported into the region for pollination interbreed with African-derived honey bees that have spread into California since the late 1980s (Pinto et al. 2005, Kono and Kohn 2015). Importantly, the Southern California population is genetically distinct from the well-characterized Africanized populations in Central and South America (Zárate et al. 2022, 2023a, 2023b).

Californian honey bees exhibit traits consistent with improved colony performance under parasite pressure. Recent work suggests differences in *Varroa destructor* (Anderson & Trueman) dynamics compared with commercial lineages (Ramos-Cuellar et al. 2022, Chong 2025, Chong-Echavez and Baer 2026), although the mechanisms underlying these patterns remain unclear and may involve tolerance, resistance, or a combination of both. Preliminary observations also suggest that Californian honey bees may show tolerance to the fungal pathogen *Nosema ceranae* (Fries) (Webb 2025). Naturally occurring tolerance has been described in other survivor populations, such as those on Fernando de Noronha Island, Brazil, which persist despite high Varroa loads (Brettell and Martin 2017), as well as in populations in South Texas (Dickey et al. 2024). In addition, Californian honey bees appear to tolerate climatic stress, including high temperatures (Leeds 2022, Allen 2025, McAfee et al. 2025). Although some Californian colonies display elevated defensiveness, which renders them unsuitable for management, others exhibit defensiveness levels comparable to commercial stocks (Zárate et al. 2023a, 2023b, Watts, personal observation). This population, therefore, provides a unique system in which evolutionary and ecological factors may favor resilient genotypes of growing interest to the beekeeping community (Zárate et al. 2023a, 2023b).

Here, we quantify the types of honey bee stocks currently maintained by beekeepers in Southern California and assess whether management practices differ among them. To address this, we conducted a survey during the annual Honey Bee Health Conference, asking attendees about the origins of the colonies they keep and the management practices they employ.

## Materials and Methods

To compare beekeeping practices used throughout Southern California, we conducted a cross-sectional survey using a questionnaire consisting of 35 questions, which is provided in Supplementary File 1 and can be downloaded at DOI: 10.5061/dryad.0k6djhbbq.

Our survey was designed to collect information across 4 focal areas: (i) sampling a population of beekeepers within regions where Californian honey bees occur naturally; (ii) characterizing the stock type they managed. To do this, we defined Californian stock as colonies headed by self-raised, openly mated queens. In contrast, commercial stock was described as colonies headed by queens that are continuosly purchased from different breeders throughout the United States. Based on these definitions, beekeepers were subsequently grouped into 3 categories: those managing exclusively commercial stock, exclusively Californian stock, or mixed stock (both Californian and commercial). We further aimed to (iii) identify differences in bee health management practices across these groups and (iv) assess variation in colony performance, including beekeeper-reported parasite prevalence, queen loss, and exposure to environmental stressors.

Data collection was primarily conducted during the annual Honey Bee Health Conference organized by the Center for Integrative Bee Research (CIBER; https://ciber.ucr.edu) and hosted at the University of California, Riverside, on September 2, 2023. The event was attended by over 120 participants, representing all major local beekeeping associations, including the Long Beach Beekeepers, the Beekeeping Association of Southern California, the Orange County Beekeepers Association, the Los Angeles County Beekeepers Association, and the San Diego Beekeeping Society. The survey allowed partial completion of the questionnaire, resulting in varying sample sizes across questions ranging from *n = *38 to *n = *93. Because this study focused on beekeeping practices within Southern California, responses from participants managing colonies outside this region were excluded prior to analysis to ensure geographic comparability. The study was conducted in accordance with University of California requirements and received Institutional Review Board (IRB) approval from UCR (HS 22-135).

### Statistical Analyses

To assess whether beekeeping practices differed among the 3 beekeeper groups (commercial, mixed, and Californian), we used a combination of parametric and nonparametric statistical tests, depending on the data type. Each survey respondent was considered an independent observational unit. For categorical variables (eg stock type, queen management strategies, parasite prevalence, and beekeeper perceptions), we used contingency tables and Pearson’s χ^2^ tests of independence. When cell counts were <5, we used Fisher’s exact tests. Pairwise group comparisons were conducted using 2 × 2 Fisher’s exact tests (or 2-proportion χ^2^ tests), and we used Holm corrections to account for multiple testing within the same dataset. Results are reported as counts (*n*/*N*), percentages (%), and 95% binomial confidence intervals (CIs) that we calculated using the Clopper–Pearson method. For multiple choice questions, denominators varied by variable (excluding non-responses), and therefore, percentages did not sum to 100%.

For the prevalence of Varroa mite and small hive beetle, we fitted logistic regression models with presence/absence as the outcome, using stock type (with commercial honey bees as the reference), beekeeper scale, and apiary size as predictors. Because some categories had low counts, apiary size was analyzed as a collapsed categorical factor. To estimate biological effect sizes, we calculated odds ratios (ORs) with 95% CIs and *P* values. All *P* values provided are 2-sided with α = 0.05.

For continuous or ordinal variables (eg annual expenditures on treatments, diagnostic tools, pest deterrents, and disease preventatives), we used Kruskal-Wallis rank-sum tests. In cases where significant effects were detected, we applied *post hoc* Dunn’s tests with Bonferroni corrections for pairwise comparisons by stock and scale, and Holm correction for comparisons by apiary size. To evaluate whether associations with stock type could be explained by operational scale or apiary size, we fitted cumulative logit models (ordinal regression with a logit link) that included both predictors.

Several survey questions allowed multiple responses (eg queen management strategies and observations of parasites and pathogens). In these cases, the frequency of each selected option was recorded and analyzed separately, resulting in response totals that exceeded the number of participants. Statistical tests were therefore based on the frequency of individual responses rather than mutually exclusive categories.

**Fig. 1.**
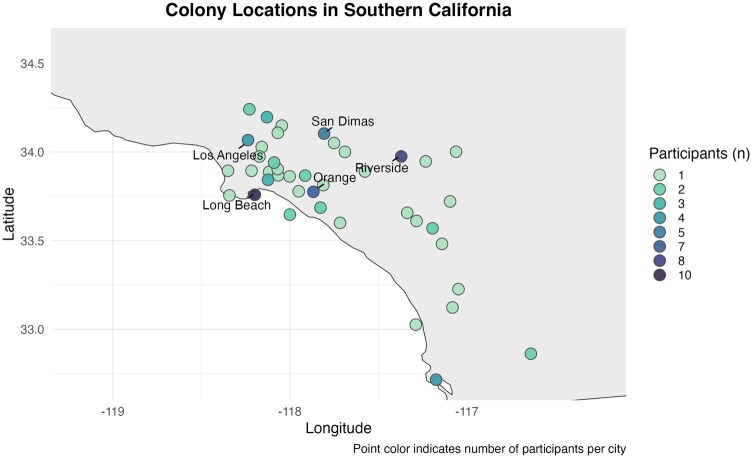
The geographic locations of honey bee colonies of 121 beekeepers that participated in the survey. Darker points indicate areas with higher numbers of respondents. Beekeepers were predominantly located in regions overlapping with the distribution of Californian bees, with many colonies maintained in highly urbanized areas around Los Angeles and San Diego.

**Fig. 2.**
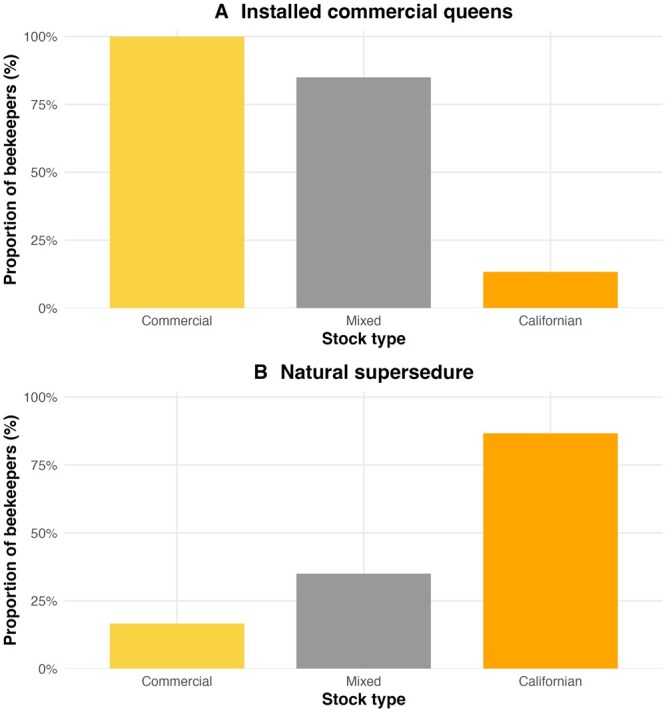
Queen management strategies reported by beekeepers managing commercial (left bars), Californian (rigth bars), or a mix of both stocks (middle bars) colonies. (A) Purchasing of queens from commercial breeders was reported by all beekeepers maintaining commercial colonies, but was significantly less common among those managing Californian or mixed stocks. (B) In contrast, beekeepers managing Californian or mixed stocks primarily allowed natural supersedure (self-replacement) of queens. Bars represent the proportion of beekeepers using each strategy.

**Fig. 3.**
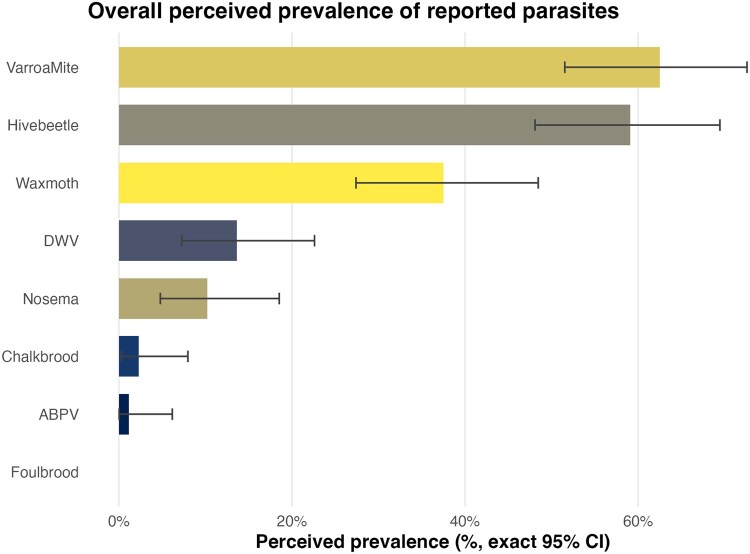
Overall perceived prevalence of reported honey bee parasites among surveyed beekeepers. Bars represent the proportion of beekeepers who reported each parasite, with error bars showing exact 95% confidence intervals. The most frequently reported parasites were Varroa mite, hive beetle, and wax moth, whereas other pathogens, such as Chalkbrood and ABPV, were rarely reported.

**Fig. 4.**
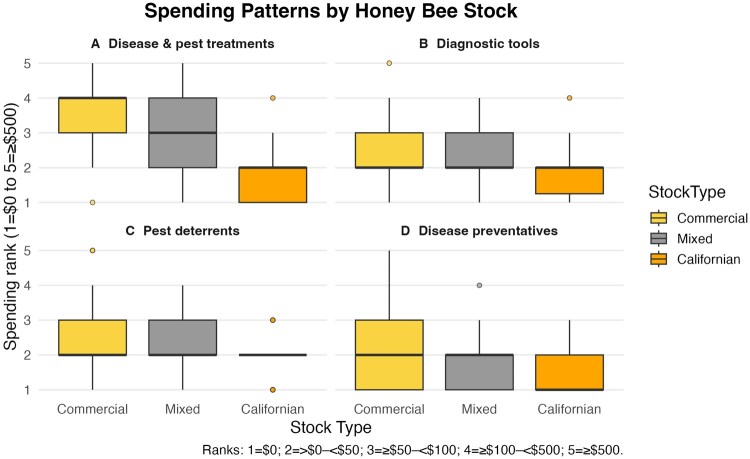
Ranked annual spending by stock type (commercial, mixed, Californian) for different beekeeping health management activities. Survey responses were ranked on a 1 to 5 scale based on spending range (1 = $0, 5 = >$500). Categories include (A) disease and pest treatments, (B) diagnostic tools and materials, (C) pest deterrents, and (D) disease preventatives. Spending ranks were coded on a 1 to 5 ordinal scale (1 = $0, 5 = ≥$500). Boxplots show median and interquartile range; whiskers denote 1.5 × IQR, and points are outliers. Statistical results are reported in Table 1.

Most statistical analyses were performed using SPSS Statistics (v29.0.1.1). Analyses related to beekeeper scale, apiary size, and cumulative logit models were conducted in R (version 2024.12.1 + 563) using the *FSA* and *ordinal* packages. Figures were generated in R: Fig. 1 (geographic distribution of colony locations) was created using the *maps* and *ggplot2* packages. Figures 2–4 are based on analyses conducted in SPSS, but plotted in R using the *ggplot2* package.

## Results

In Question 2, survey participants self-identified their operation scale. The majority classified themselves as hobbyists (*n = *75; 80.6%), while 6 (6.5%) identified as sideliners and 12 (12.9%) as commercial beekeepers. Apiary sizes ranged from 1 to 999 colonies (Question 4). Among those beekeepers actively managing colonies, most maintained small apiaries: 32 (34.4%) managed 1 to 2 colonies, 17 (18.3%) maintained 3 to 5 colonies, and 11 (11.8%) reported 6 to 10 colonies. Beekeepers with larger apiaries were less frequent, with 12 respondents (12.9%) maintaining 11 to 99 colonies and 7 (7.5%) managing 100 to 999 colonies. These colonies were located (Question 5) predominantly in Southern California (*n = *83; 93.3%) (Fig. 1), with fewer in Northern California (*n = *1; 1.1%), other U.S. states (*n = *2; 2.2%), or outside the United States (*n = *3; 3.4%). As described in Materials and Methods, responses from participants managing colonies outside Southern California (*n = *6) were excluded from subsequent analyses. To address the second objective, beekeepers were classified by the origin of their stock (Question 6). Among the 85 respondents who provided responses, 30 (35.3%) managed exclusively “commercial stock,” 25 (29.4%) managed exclusively “Californian stock,” and 30 (35.3%) maintained “mixed stock.” These categories were used for all subsequent analyses.

### Queen Management Practices

We next examined queen loss management (Question 7.2). A total of 51 beekeepers reported 1 or more strategies. The most common responses were requeening with purchased queens (30/51, 58.8%) or allowing natural supersedure, which is the process by which a honey bee colony replaces its queen on its own, usually when the existing queen becomes old, weak, or less productive (26/51, 51%). Fewer beekeepers combined queen-less with queen-right colonies (17/51, 33.33%) or allowed colonies to die (6/51, 15.8%). Moreover, significant differences were observed among the 3 groups of beekeepers. All beekeepers managing commercial stock (10/10, 100%) reported requeening with purchased queens, whereas only 1 (10%) allowed supersedure. In contrast, most of those managing Californian stock relied on supersedure (17/19, 89.5%), with only 2/19 (10.5%) reporting the use of purchased queens. Beekeepers managing mixed stock more frequently requeened with purchased queens (18/22, 81.8%); roughly one-third (8/22, 36.4%) also allowed colonies to supersede naturally. Fisher’s exact tests revealed significant differences among the 3 beekeeper groups in their use of purchased queens (*P < *0.001) and natural supersedure (*P < *0.001). In contrast, those managing Californian stock were more likely to allow supersedure than either commercial (Fisher’s exact test, *P < *0.001) or mixed stocks (Fisher’s exact test, *P =* 0.002).

Beekeepers also reported their requeening frequency (Question 7.3.1; *N = *41). The majority requeened once a year (14/41, 34.1%) or every 2 years (11/41, 26.8%), with fewer requeening every 6 months (2/41, 4.9%) or only when needed (3/41, 7.3%). Requeening frequencies differed significantly among stock types (Kruskal-Wallis H(2) = 13.1, *P =* 0.001). Dunn’s *post hoc* tests showed that beekeepers with Californian stocks requeened less frequently than those with mixed stocks (*P =* 0.001). No significant differences were detected between Californian and commercial stocks (*P =* 0.370) or between commercial and mixed stocks (*P =* 0.200). Furthermore, an ordinal regression confirmed an overall effect of stock type on requeening frequency (Wald χ^2^ = 13.4, df = 2, *P =* 0.001).

### Disease Management

#### Monitoring Frequency

We next analyzed the responses concerning disease management (Question 10). Most respondents reported evaluating their colonies for the presence of parasites and pathogens. However, the frequency of evaluations differed significantly among the responding beekeeper groups (commercial: *n = *29; mixed: *n = *27; Californian: *n = *23, Fisher’s exact test, *P =* 0.028). *Post hoc* tests indicated that Californian beekeepers evaluated their hives less frequently than those managing mixed stock (Holm-adj *P =* 0.024).

#### Observed Parasites and Pathogens

In Question 9, most respondents (83%, 73/88) reported the perceived presence of at least 1 parasite or pathogen. The most frequently reported parasite was Varroa mite (*Varroa destructo*r Anderson and Trueman; *n = *55, 62.5%, 95% CI: 51.5 to 72.6), the small hive beetle (*Aethina tumida* Murray; *n = *52, 59.1%, 95% CI: 48.1 to 69.5), and the greater wax moth (*Galleria mellonella* Linnaeus; *n = *33, 37.5%, 95% CI: 27.4 to 48.5). Less frequently reported pathogens were deformed wing virus (DWV; *Iflavirus aladeformis*; *n = *12, 13.6%, 95% CI: 7.2 to 22.6) and *Nosema* spp. (*n = *9, 10.2%, 95% CI: 4.8 to 18.5). Rarely observed were chalkbrood (*Ascosphaera apis* Olive & Spiltoir; *n = *2, 2.3%, 95% CI: 0.3 to 8.0) and acute bee paralysis virus (ABPV; *Aparavirus apisacutum*; *n = *1, 1.1%, 95% CI: 0.0 to 6.2). Notably, European or American foulbrood (*Melissococcus plutonius/Paenibacillus larvae* White) was not reported by any of the beekeepers (Fig. 3A).

### Group Comparisons

We also compared perceptions of parasites among the 3 beekeeper groups (*n = *81). Reports of Varroa mites did not differ significantly among groups (χ^2^ = 4.47, df = 2, *P =* 0.107). Varroa was reported by 21/29 (72.4%) beekeepers managing commercial stock, 20/28 (71.4%) with mixed stock, and 11/24 (45.8%) with Californian stock. Similarly, reports of small hive beetles showed no significant differences (χ^2^ = 2.54, df = 2, *P =* 0.281), with 16/29 (55.2%) of commercial, 16/28 (57.1%) of mixed, and 18/24 (75.0%) of Californian stock beekeepers affected. Greater wax moths were reported at comparable levels (χ^2^ = 1.99, df = 2, *P =* 0.370) by 13/29 (44.8%) of commercial, 8/28 (28.6%) of mixed, and 10/24 (41.7%) of Californian stock beekeepers. Reports of deformed wing virus (DWV) also did not differ significantly (Fisher’s exact, *P =* 0.231), being noted by 4/29 (13.8%) commercial, 6/28 (21.4%) mixed, and 1/24 (4.2%) Californian stock beekeepers. Other pathogens were rarely reported: ABPV (Fisher’s exact, *P =* 1.000) was mentioned only by 2/28 (3.6%) mixed stock beekeepers, and chalkbrood (Fisher’s exact, *P =* 0.328) by 2/28 (7.1%) mixed stock beekeepers.

*Nosema* spp. infestations differed among groups (Fisher’s exact, *P =* 0.039), being reported by 2/29 (6.9%) commercial and 6/28 (21.4%) mixed stock beekeepers, but by none of the Californian stock beekeepers. However, none of the pairwise *post hoc* comparisons reached significance.

We also assessed parasite burden, defined as the number of parasite taxa reported per beekeeper (range: 0 to 8). Richness did not differ significantly among the 3 groups (Kruskal-Wallis H(2) = 2.75, *P =* 0.250).

Finally, logistic regression models revealed no statistically significant differences in parasite prevalence between stocks. For Varroa mites, odds of reporting infestation were lower in Californian compared to commercial stock (OR = 0.28, 95% CI: 0.09 to 1.08; χ^2^ = 3.39, df = 1, *P =* 0.066). Mixed stock did not differ from commercial stock (OR = 0.79, 95% CI: 0.23 to 2.74, *P =* 0.708). Odds of reporting small hive beetles were higher among beekeepers managing Californian stock (OR = 2.52, 95% CI: 0.73 to 8.70; χ^2^ = 2.13, df = 1, *P =* 0.144), though this effect was not significant. All other pairwise comparisons were non-significant (all *P* > 0.47).

#### Use of Diagnostic Tools

In Question 11, beekeepers were asked about the diagnostic tools they use to quantify Varroa infestations. Alcohol washes were the predominant method among those managing commercial (17/29, 58.6%) and mixed stock (17/27, 63.0%). Sugar shakes were also widely employed, reported by 13/29 (44.8%) of commercial and 16/27 (59.3%) of mixed stock beekeepers. In contrast, diagnostic practices differed among beekeepers managing Californian stock. Only 1/23 (4.3%) reported using alcohol washes, whereas 8/23 (34.8%) relied on sugar shakes, and an equal proportion (8/23, 34.8%) indicated that they did not perform Varroa checks at all. Statistical analysis confirmed significant differences in alcohol wash usage (Fisher’s exact, *P < *0.001), with pairwise *post hoc* comparisons showing significantly lower use among beekeepers managing Californian bees compared to both commercial and mixed stock beekeepers (Holm-adjusted *P < *0.001). Reporting of no diagnostic checks was also significantly more frequent among beekeepers managing Californian bees compared to those keeping commercial stock (Holm-adjusted *P =* 0.020).

#### Estimated Costs for Hive Management

We also asked beekeepers to report their approximate financial expenditures on colony management (Questions 13 to 16). (Q13, *n = *78). The costs for treatments against parasites such as Apivar, fumagillin, and formic acid differed significantly between the 3 groups of beekeepers (Kruskal-Wallis H(2) = 23.5, *P < *0.001). Beekeepers managing Californian stock reported significantly lower spending compared with those managing commercial (*Z* = 4.79, *P < *0.001) or mixed stock (*Z* = 3.30, *P =* 0.003) (Table 1; Fig. 4A).

**Table 1.** Differences in annual beekeeping expenditures by stock type (commercial, mixed, Californian) across spending categories (Q13-Q16)

Survey question	Kruskal-Wallis test		Dunn’s *post hoc* test		
	*H* (df)	*P*-value	Pairwise comparison	*Z*-value	*P* adj. (Bonferroni)
**Q13: Diseases and Pest Treatments**	23.5 (2)	<0.001*	Commercial ↑ Californian	4.79	<0.001*
			Mixed ↑ Californian	3.30	0.003**
			Commercial vs. Mixed	1.52	0.38
**Q14: Diagnostic Tools**	6.00 (2)	0.0499*	Commercial ↑ Californian	2.45	0.043***
			Mixed vs. Californian	1.26	0.628
			Commercial vs. Mixed	1.24	0.649
**Q15: Pest Deterrents**	9.17 (2)	0.0102**	Commercial ↑ Californian	2.84	0.0136***
			Mixed ↑ Californian	2.46	0.041***
			Commercial vs. Mixed	0.35	1.000
**Q16: Disease Preventatives**	10.3 (2)	0.0059**	Commercial ↑ Californian	3.20	0.0041**
			Mixed vs. Californian	1.89	0.176
			Commercial vs. Mixed	1.36	0.526

The Kruskal-Wallis test was used to assess overall differences in ordinal spending ranks. *Post hoc* comparisons were performed using Dunn’s tests with Bonferroni correction. Arrows indicate the direction of the pairwise effect (A ↑ B = A had a higher spending rank than B). Bold indicates adjusted *P < *0.05; asterisks denote significance levels:

* *P < *0.001,

** *P < *0.01,

*** *P < *0.05.

Expenditures for diagnostic tools and materials (Q14, *n = *75), such as sugar shakes, alcohol washes, virus and Nosema tests, and microscopes, differed among the beekeeper groups. This difference was statistically significant (H(2) = 6.0, *P =* 0.0499). *Post hoc* tests revealed lower expenditures among beekeepers managing Californian bees than among those with commercial stock (*Z* = 2.45, *P =* 0.043); all other pairwise comparisons were not significant (Table 1; Fig. 4B).

Spending on pest deterrents (Q15, *n = *78) included products to manage ants or hive beetles, and this spending also differed significantly among the 3 beekeeper groups (H(2) = 9.17, *P =* 0.010). Beekeepers managing Californian stock reported lower expenses than those managing commercial (*Z* = 2.84, *P =* 0.014) or mixed stock (*Z* = 2.46, *P =* 0.041) (Table 1; Fig. 4C).

Finally, costs for disease prevention measures, such as probiotics, supplements, or vitamins (Q16, *n = *77), also differed significantly among the 3 groups (H(2) = 10.3, *P =* 0.006). *Post hoc* tests indicated that Californian beekeepers reported lower expenditures compared with those keeping commercial stock (*Z* = 3.20, *P =* 0.004). At the same time, no significant differences were found between those maintaining commercial and mixed stock (*Z* = 1.36, *P =* 0.526) or between Californian and mixed stock (*Z* = 1.89, *P =* 0.176) (Table 1; Fig. 4D).

#### Beekeeping Costs by Operation Scale and Apiary Size

When expenditures were compared across beekeepers operating scale (hobbyist, sideliner, commercial), Kruskal-Wallis tests showed significant differences only for pest deterrents (Q15; H(2) = 13.82, *P =* 0.001), while no significant variation was observed for disease treatments (Q13; Kruskal-Wallis H(2) = 2.47, *P =* 0.291), diagnostic tools (Q14; H(2) = 5.90, *P =* 0.053), or disease preventives (Q16; H(2) = 3.69, *P =* 0.158). *Post hoc* test indicated that sideliners spent more on pest deterrents than either hobbyists (*Z* = 3.72, *P =* 0.001) or commercial beekeepers (*Z* = 2.78, *P =* 0.016). In contrast, differences between hobbyists and commercial beekeepers were not significant (*Z* = 0.37, *P =* 1.00). To test whether these differences persisted after accounting for stock type and beekeeper scale, we fitted a cumulative logit model that included both predictors. These models confirmed persistent effects of stock type on spending patterns (Q13: *P < *0.001; Q14: *P =* 0.030; Q15: *P =* 0.004; Q16: *P =* 0.005).

Expenditures also varied by apiary size. Kruskal-Wallis tests detected significant differences in spending on disease treatments (Q13; H(5) = 15.7, *P =* 0.008) and pest deterrents (Q15; H(5) = 14.7, *P =* 0.012), but not for diagnostic tools (Q14; H(5) = 9.60, *P =* 0.087) or disease preventives (Q16; H(5) = 7.88, *P =* 0.163). *Post hoc* tests showed that beekeepers with 1 to 2 colonies spent significantly less on disease treatments than those with 100 to 999 colonies (*Z* = −3.06, *P =* 0.033), whereas no pairwise comparisons were significant for pest deterrents. Because expenditures may also depend on stock type, we fitted a cumulative logit model that incorporates both apiary size and stock type. The model confirmed independent effects of both predictors, with lower spending reported for Californian stock and higher expenditures in larger apiaries (Q13: *P < *0.001; Q14: *P =* 0.030; Q15: *P =* 0.004; Q16: *P =* 0.004).

#### Responses of Beekeepers Using Both Stock

We also analyzed responses from beekeepers who managed both commercial and Californian colonies to assess perceived differences between their stock types (Questions 6.1 to 6.3).

For perceived disease and parasite differences (Q6.1, *n = *30), responses differed significantly (χ^2^ = 8.60, df = 2, *P =* 0.014; Holm-adjusted *P =* 0.014). A majority of these beekeepers (16/30, 53.3%) reported that commercial colonies harbored more diseases than Californian colonies, while (3/30, 10.0%) indicated the opposite, and 11/30 (36.7%) reported no difference. Pairwise *post hoc* comparison confirmed that commercial colonies were significantly more often reported as harboring more diseases than Californian colonies (*P* adj = 0.003), whereas no other contrasts were significant.

For heat tolerance (Q6.2, *n = *28), most respondents reported no differences between stock types (18/28, 64.3%). In contrast, (8/28, 28.6%) respondents reported greater heat tolerance in Californian colonies and (2/28, 7.1%) in commercial colonies. Responses also differed significantly (χ^2^ = 14.00, df = 2, *P < *0.001; Holm-adjusted *P =* 0.002). *Post hoc* analysis showed that “no difference” responses were significantly over-represented (*P* adj = 0.014), while reports that “commercial colonies tolerate heat more” were significantly under-represented (*P* adj = 0.032).

For honey production (Q6.3, *n = *27), responses did not differ significantly (χ^2^ = 1.90, df = 2, *P =* 0.387; Holm-adjusted *P =* 0.405). Most beekeepers (19/27, 70.4%) reported no difference in honey production between their commercial and Californian colonies, while 3/27 (11.1%) considered commercial colonies superior producers and 5/27 (18.5%) favored Californian colonies. No pairwise *post hoc* comparison reached significance.

## Discussion

Ongoing declines in honey bee health have triggered a growing interest in management strategies that reduce chemical interventions and emphasize the use of locally adapted stocks (Guichard et al. 2020, Smith et al. 2023, Gray et al. 2024, Chong-Echavez and Baer 2026). In Southern California, this shift is reflected in the widespread use of so-called “Californian” honey bee colonies, which arise from open mating and local adaptation. Our survey was designed to capture this phenomenon by sampling beekeepers in areas where Californian bees occur naturally, classifying their stock into commercial, Californian, or mixed categories (ie beekeepers maintaining both commercial and Californian colonies), and comparing how management strategies and perceptions vary across beekeeper groups.

### Stock Adaptation and Management Shifts

The results reveal that Californian stock is already well integrated into regional beekeeping. More than 60% of respondents reported keeping Californian colonies either exclusively or in combination with commercial colonies. This widespread adoption likely reflects the presence of locally adapted, admixed populations that carry African ancestry (Pinto et al. 2005, Kono and Kohn 2015, Calfee et al. 2020, Zárate et al. 2022). Beekeepers may also recognize adaptive traits in these populations, such as disease resistance (Geffre et al. 2023, Chong 2025, Chong-Echavez and Baer 2026) and thermal resilience (Allen 2025, McAfee et al. 2025).

Beekeepers managing Californian colonies reported fewer inspections, less use of diagnostic tools, and lower expenditures on treatments and requeening than those managing commercial or mixed stocks. This pattern may reflect both biological traits and differences in management strategies among Californian honey bees. Reduced inspection frequency can be a deliberate strategy to minimize disturbance, which affects brood thermoregulation (Cook et al. 2022, Hossain et al. 2025), rather than a sign of neglect. Importantly, these reduced management inputs did not correspond to higher perceived disease levels. These findings may indicate that Californian colonies can function under lower management intensity, but reduced monitoring may also limit the detection of disease events. These observations do not allow a clear distinction between tolerance and resistance mechanisms. In contrast, beekeepers who managed both commercial and Californian colonies tended to follow practices closer to conventional management, with more frequent inspections and greater use of diagnostic tools. This suggests that management strategies are shaped not only by stock type but also by individual beekeeper preferences and experience (Kulhanek et al. 2017, Maciejovsky et al. 2023).

### Perceptions and Health Outcomes

Beekeepers managing both commercial and Californian colonies provided valuable comparative insights. Commercial colonies were often described as more disease-prone than Californian colonies. However, quantitative analyses did not reveal statistically significant differences in parasite prevalence among stock types. This discrepancy underscores the distinction between beekeeper perceptions of colony health and measured disease outcomes, which may be influenced by differences in monitoring intensity, treatment practices, or the visibility of specific pests. Respondents mainly referred to visible pests such as *Varroa destructor* and small hive beetles, whereas pathogens requiring laboratory diagnostics were rarely mentioned. This highlights both the value and the limitations of perception-based data, as beekeepers can readily detect conspicuous problems, but pathogens that often require laboratory confirmation (eg viruses and *Nosema* spp.) are less likely to be detected and reported (Cleary and Szalanski 2022).

These perceptions are consistent with tolerance mechanisms, in which colonies maintain performance despite infection rather than resistance, which reduces infection intensity (Mattila and Seeley 2007, Locke 2016, Brettell and Martin 2017, Guzman-Novoa et al. 2024). Tolerant colonies can sustain background levels of pathogen without exhibiting overt signs of disease. This can lower management demands, but it also raises questions about pathogen persistence in the environment. Previous studies suggest that tolerant colonies may act as reservoirs, potentially facilitating spillover to other managed colonies or wild pollinators (Manley et al. 2015, Sokolov et al. 2025). However, tolerance does not always lead to increased transmission. For example, viral loads in feral and managed colonies in Southern California were similar (Geffre et al. 2021), suggesting that tolerant populations do not necessarily amplify pathogen spread. Understanding when tolerance leads to pathogen spread versus stable background infection dynamics will require targeted epidemiological studies. This study relies on self-reported data. Observer bias is therefore possible, and responses may be influenced by experience or expectations. Future work should combine beekeeper surveys with molecular and standardized diagnostic protocols to better link perceptions with biological outcomes. The extent to which these patterns reflect tolerance, resistance, or their interaction remains unclear and represents an important priority for future experimental research.

### Economic and Practical Implications

The management differences reported by beekeepers also have clear economic consequences. Beekeepers managing Californian colonies reported lower expenditures on treatments, diagnostics, and requeening compared with those managing commercial or mixed stocks. These reductions likely reflect reduced management intensity and a perceived decrease in disease burden (Degrandi-Hoffman et al. 2019). However, these expenditure differences may reflect management strategies and perceived needs rather than underlying biological differences in colony health. This is particularly relevant given the rising costs of queen replacement (Rucker et al. 2019) and other apicultural supplies (Tubene et al. 2023).

For hobbyist beekeepers, who represented the majority of respondents, lower recurring costs can reduce financial barriers to participation and make beekeeping more economically sustainable. Hobbyists play an increasingly important role in maintaining local pollination networks and conserving genetic diversity, and they may also be more willing to adopt locally adapted stocks and alternative management strategies (Maciejovsky et al. 2023). Reduced reliance on chemical treatments also aligns with growing consumer demand for sustainable and low-input honey production, potentially creating opportunities for market differentiation (Underwood et al. 2023).

### Future Research Opportunities

Our findings highlight both the potential and the current knowledge gaps associated with the widespread use of Californian honey bees. Although these locally adapted, admixed populations appear to perform well under lower management intensity, further research is required to better understand their life-history traits and long-term value for pollination services. Key questions include whether these stocks differ in colony growth dynamics, seasonal survival, overwintering success, reproductive output, and pollination efficiency relative to commercially managed lines.

Addressing these questions will be essential to determine their long-term role in sustainable apiculture and their potential integration into structured breeding programs. As adoption increases, management-related traits beyond productivity and disease tolerance, such as defensiveness, swarming tendency, and colony stability, will require systematic evaluation. Incorporating standardized measures of colony survival and productivity across seasons would also strengthen comparisons among stock types and management systems.

Equally important is the need to validate self-reported differences using standardized protocols and genetic tools. Applying established frameworks such as COLOSS survey methodologies and molecular diagnostics to both managed and feral populations would help clarify pathogen dynamics, management outcomes, and genetic backgrounds (Pirk et al. 2013, Wallberg et al. 2014, Brodschneider et al. 2018). Expanding this work into commercial operations would further test whether the reported cost and health benefits persist under large-scale, migratory management. Future surveys could also collect continuous expenditure data and precise colony counts to enable standardized cost-per-colony metrics across operation sizes.

Citizen science initiatives offer a promising path forward (Brodschneider et al. 2019). Coordinated monitoring efforts within the Southern California beekeeping community could facilitate longitudinal datasets across diverse environments, providing robust empirical evidence to guide adaptive management and future breeding strategies.

## Conclusion

Californian honey bees are widely kept across Southern California. Beekeepers perceive them as healthier and less expensive to manage than commercial stocks. These findings emphasize that reported differences in colony health should be interpreted in the context of management practices, monitoring intensity, and beekeeper perceptions. These reported advantages likely reflect a combination of local genetic adaptation, management strategies, and beekeeper perceptions. Important uncertainties remain, including pathogen dynamics and large-scale performance. Targeted research addressing these questions will determine whether Californian bees can be effectively integrated into broader apicultural practice and whether their traits can inform breeding strategies for sustainable beekeeping under future environmental pressures.

## References

[toag099-B1] Al Naggar Y , Baer B. 2019. Consequences of a short time exposure to a sublethal dose of Flupyradifurone (Sivanto) pesticide early in life on survival and immunity in the honeybee (*Apis mellifera*). Sci. Rep. 9:19753. 10.1038/s41598-019-56224-1 31874994 PMC6930273

[toag099-B2] Allen C. 2025. The genetic secrets of survivor bees. BeeCraft 107:28–30.

[toag099-B3] Bloch G , Francoy TM, Wachtel I, et al 2010. Industrial apiculture in the Jordan valley during Biblical times with Anatolian honeybees. Proc. Natl. Acad. Sci. USA 107:11240–11244. 10.1073/pnas.1003265107 20534519 PMC2895135

[toag099-B4] Branchiccela B , Aguirre C, Parra G, et al 2014. Genetic changes in *Apis mellifera* after 40 years of Africanization. Apidologie 45:752–756.

[toag099-B5] Brettell LE , Martin SJ. 2017. Oldest Varroa tolerant honey bee population provides insight into the origins of the global decline of honey bees. Sci. Rep. 7:45953. 10.1038/srep45953 28393875 PMC5385554

[toag099-B6] Brodschneider R , Gratzer K, Kalcher-Sommersguter E, et al 2019. A citizen science supported study on seasonal diversity and monoflorality of pollen collected by honey bees in Austria. Sci. Rep. 9:16633. 31719621 10.1038/s41598-019-53016-5 PMC6851371

[toag099-B7] Brodschneider R , Gray A, Adjlane N, et al 2018. Multi-country loss rates of honey bee colonies during winter 2016/2017 from the COLOSS survey. J. Apic. Res. 57:452–457.

[toag099-B8] Calfee E , Agra MN, Palacio MA, et al 2020. Selection and hybridization shaped the rapid spread of African honey bee ancestry in the Americas. PLoS Genet. 16:e1009038. 33075065 10.1371/journal.pgen.1009038 PMC7595643

[toag099-B9] Chong G. 2025. Survivor bees and Varroa: lessons from nature. BeeCraft 107:14–16.

[toag099-B4327913] Chong-Echavez G , Baer B. 2026. Varroa mite resistance in a hybrid honey bee (*Apis mellifera*) population in Southern California. Sci. Rep. 16:10952. 10.1038/s41598-026-45759-9 41896641 PMC13039435

[toag099-B10] Cleary D , Szalanski AL. 2022. Molecular diagnostic survey of honey bee, *Apis mellifera* L., pathogens and parasites from Arkansas, USA. J. Apic. Sci. 66:149–158.

[toag099-B11] Cobey S , Sheppard WS, Tarpy DR. 2011. Status of breeding practices and genetic diversity in domestic U.S. honey bees. In: Sammataro D, Yoder JA, editors. Honey bee colony health. CRC Press. p. 39–49.

[toag099-B12] Cook D , Tarlinton B, McGree JM, et al 2022. Temperature sensing and honey bee colony strength. J. Econ. Entomol. 115:715–723. 35522232 10.1093/jee/toac034 PMC9175291

[toag099-B13] Degrandi-Hoffman G , Graham H, Ahumada F, et al 2019. The economics of honey bee (Hymenoptera: Apidae) management and overwintering strategies for colonies used to pollinate almonds. J. Econ. Entomol. 112:2524–2533. 31504631 10.1093/jee/toz213

[toag099-B14] Delaplane KS , Given JK, Menz J, et al 2021. Colony fitness increases in the honey bee at queen mating frequencies higher than genetic diversity asymptote. Behav. Ecol. Sociobiol. 75:138. 10.1007/s00265-021-03065-6

[toag099-B15] Dickey M , Whilden M, Ellis JT, et al 2024. Comparative prevalence of *Nosema ceranae* infection between wild and managed honey bee (*Apis mellifera*) colonies in South Texas. Apidologie 55:1–14.

[toag099-B16] Geffre A , Travis D, Kohn J, et al 2023. Preliminary analysis shows that feral and managed honey bees in Southern California have similar levels of viral pathogens. J. Apic. Res. 62:485–487. 10.1080/00218839.2021.2001209

[toag099-B17] Goulson D , Nicholls E, Botías C, et al 2015. Bee declines driven by combined stress from parasites, pesticides, and lack of flowers. Science 347:1255957. 25721506 10.1126/science.1255957

[toag099-B18] Gray D , Goslee S, Kammerer M, et al 2024. Effective pest management approaches can mitigate honey bee (*Apis mellifera*) colony winter loss across a range of weather conditions in small-scale, stationary apiaries. J. Insect. Sci. 24:15. 10.1093/jisesa/ieae043 PMC11132132 38805654

[toag099-B19] Guichard M , Dietemann V, Neuditschko M, et al 2020. Advances and perspectives in selecting resistance traits against the parasitic mite *Varroa destructor* in honey bees. Genet. Sel. Evol. 52:71. 33246402 10.1186/s12711-020-00591-1 PMC7694340

[toag099-B20] Guzman-Novoa E , Corona M, Alburaki M, et al 2024. Honey bee populations surviving *Varroa destructor* parasitism in Latin America and their mechanisms of resistance. Front. Ecol. Evol. 12:1434490.

[toag099-B21] Hossain MS , Faloutsos C, Baer B, et al 2025. Principled mining, forecasting and monitoring of honeybee time series with EBV+. ACM Trans. Knowl. Discov. Data 19:1–30. 10.1145/3719014

[toag099-B22] Kono Y , Kohn JR. 2015. Range and frequency of Africanized honey bees in California (USA). PLoS One 10:e0137407. 26361047 10.1371/journal.pone.0137407 PMC4567290

[toag099-B23] Kulhanek K , Steinhauer N, Rennich K, et al 2017. A national survey of managed honey bee 2015–2016 annual colony losses in the USA. J. Apic. Res. 56:328–340.

[toag099-B24] Lamas ZS , Chen Y, Evans JD. 2024. Case report: emerging losses of managed honey bee colonies. Biology (Basel). 13:117. 38392335 10.3390/biology13020117 PMC10887003

[toag099-B25] Le Conte Y , Navajas M. 2008. Climate change: impact on honey bee populations and diseases. Rev. Sci. Tech. 27:485–97, 499–510. 18819674

[toag099-B26] Leeds A. 2022. Thermotolerance of feral and managed honey bees (*Apis mellifera*) in Southern California [master’s thesis]. University of California, San Diego.

[toag099-B27] Locke B. 2016. Inheritance of reduced Varroa mite reproductive success in reciprocal crosses of mite-resistant and mite-susceptible honey bees (*Apis mellifera*). Apidologie 47:583–588.

[toag099-B28] Maciejovsky B , Baer-Imhoof B, Baer B. 2023. Hobbyist beekeepers and their importance for future beekeeping and food production. Bee World 100:80–83.

[toag099-B29] Manley R , Boots M, Wilfert L. 2015. Emerging viral disease risk to pollinating insects: ecological, evolutionary and anthropogenic factors. J. Appl. Ecol. 52:331–340. 25954053 10.1111/1365-2664.12385 PMC4415536

[toag099-B30] Mattila HR , Seeley TD. 2007. Genetic diversity in honey bee colonies enhances productivity and fitness Science. 317:362–364. 17641199 10.1126/science.1143046

[toag099-B31] McAfee A , Metz BN, Connor P, et al 2025. Factors affecting heat resilience of drone honey bees (*Apis mellifera*) and their sperm. PLoS One 20:e0317672. 39919074 10.1371/journal.pone.0317672 PMC11805398

[toag099-B32] Naug D. 2009. Nutritional stress due to habitat loss may explain recent honeybee colony collapses. Biol. Conserv. 142:2369–2372.

[toag099-B33] Pinto MA , Rubink WL, Patton JC, et al 2005. Africanization in the United States: replacement of feral European honeybees (*Apis mellifera* L.) by an African hybrid swarm. Genetics 170:1653–1665. 15937139 10.1534/genetics.104.035030 PMC1449774

[toag099-B34] Pirk CWW , de Miranda JR, Kramer M, et al 2013. Statistical guidelines for *Apis mellifera* research. J. Apic. Res. 52:1–24.

[toag099-B35] Ramos-Cuellar AK , De la Mora A, Contreras-Escareño F, et al 2022. Genotype, but not climate, affects the resistance of honey bees (*Apis mellifera*) to viral infections and to the mite *Varroa destructor*. Vet. Sci. 9:358. 35878375 10.3390/vetsci9070358 PMC9320602

[toag099-B36] Roffet-Salque M , Regert M, Evershed RP, et al 2015. Widespread exploitation of the honeybee by early Neolithic farmers. Nature 527:226–230. 26560301 10.1038/nature15757

[toag099-B37] Rucker RR , Thurman WN, Burgett M. 2019. Colony collapse and the consequences of bee disease: market adaptation to environmental change. J. Assoc. Environ. Resour. Econ. 6:927–960.

[toag099-B38] Schneider SS , DeGrandi-Hoffman G, Smith DR. 2004. The African honey bee: factors contributing to a successful biological invasion. Annu. Rev. Entomol. 49:351–376. 14651468 10.1146/annurev.ento.49.061802.123359

[toag099-B39] Tosi S , Nieh A, Brandt M, et al 2021. Long-term field-realistic exposure to a next-generation pesticide, flupyradifurone, impairs honey bee behaviour and survival. Commun. Biol. 4:805. 34183763 10.1038/s42003-021-02336-2 PMC8238954

[toag099-B40] Smith S , Moro A, McCormack GP. 2023. Exploring a potential avenue for beekeeping in Ireland: safeguarding locally adapted honeybees for breeding Varroa-resistant lines. Insects 14:827. 37887838 10.3390/insects14100827 PMC10607453

[toag099-B41] Sokolov NA , Boots M, Bartlett LJ. 2025. Avoiding the tragedies of parasite tolerance in Darwinian beekeeping. Proc. Biol. Sci. 292: 20242433. 39904384 10.1098/rspb.2024.2433 PMC11793967

[toag099-B42] Spleen AM , Lengerich EJ, Rennich K, et al; for the Bee Informed Partnership. 2013. A national survey of managed honey bee 2011–12 winter colony losses in the United States: results from the Bee Informed Partnership. J. Apic. Res. 52:44–53.

[toag099-B43] Tarpy DR , Seeley TD. 2006. Lower disease infections in honeybee (*Apis mellifera*) colonies headed by polyandrous vs. monandrous queens. Naturwissenschaften 93:195–199. 16518641 10.1007/s00114-006-0091-4

[toag099-B44] Tarpy DR , Vanengelsdorp D, Pettis JS. 2013. Genetic diversity affects colony survivorship in commercial honey bee colonies. Naturwissenschaften 100:723–728. 23728203 10.1007/s00114-013-1065-y

[toag099-B45] Tubene S , Kulhanek K, Rennich K, et al 2023. Best management practices increase profitability of small-scale US beekeeping operations. J. Econ. Entomol. 116:47–55. 36373593 10.1093/jee/toac174

[toag099-B46] Underwood RM , Lawrence BL, Turley NE, et al 2023. A longitudinal experiment demonstrates that honey bee colonies managed organically are as healthy and productive as those managed conventionally. Sci. Rep. 13:6072. 37055462 10.1038/s41598-023-32824-w PMC10100614

[toag099-B47] vanEngelsdorp D , Evans JD, Saegerman C, et al 2009. Colony collapse disorder: a descriptive study. PLoS One 4:e6481. 19649264 10.1371/journal.pone.0006481 PMC2715894

[toag099-B48] Vasiliev D , Greenwood S. 2021. The role of climate change in pollinator decline across the Northern Hemisphere is underestimated. Sci. Total Environ. 775:145788. 33618305 10.1016/j.scitotenv.2021.145788

[toag099-B49] Wallberg A , Han F, Wellhagen G, et al 2014. A worldwide survey of genome sequence variation provides insight into the evolutionary history of the honeybee *Apis mellifera*. Nat. Genet. 46:1081–1088. 25151355 10.1038/ng.3077

[toag099-B50] Webb J. 2025. Survivor bees vs. disease. BeeCraft 107:14–15.

[toag099-B51] Zárate D , Lima TG, Poole JD, et al 2022. Admixture in Africanized honey bees (*Apis mellifera)* from Panamá to San Diego, California (USA). Ecol. Evol. 12:e8580. 35222958 10.1002/ece3.8580 PMC8844128

[toag099-B52] Zárate D , Mukogawa B, Kohn J, et al 2023a. Seasonal variation in defense behavior in European and scutellata-hybrid honey bees (*Apis mellifera*) in Southern California. Sci. Rep. 13:12790. 37550348 10.1038/s41598-023-38153-2 PMC10406949

[toag099-B53] Zárate D , Travis D, Geffre A, et al 2023b. Three decades of “Africanized” honey bees in California. Calif. Agr. 77:15–20.

